# New molecular insights into the tyrosyl-tRNA synthase inhibitors: CoMFA, CoMSIA analyses and molecular docking studies

**DOI:** 10.1038/s41598-017-10618-1

**Published:** 2017-09-14

**Authors:** Shengrong Li, Jilin Fan, Chengkang Peng, Yiqun Chang, Lianxia Guo, Jinsong Hou, Miaoqi Huang, Biyuan Wu, Junxia Zheng, Longxin Lin, Gaokeng Xiao, Weimin Chen, Guochao Liao, Jialiang Guo, Pinghua Sun

**Affiliations:** 1 Guangdong Province Key Laboratory of Pharmacodynamic Constituents of TCM and New Drugs Research, College of Pharmacy, Jinan University, Guangzhou, 510632 P.R. China; 2 School of Stomatology and Medicine, Foshan University, Foshan, 528000 P.R. China; 3 School of Chemical Engineering and Light Industry, Guangdong University of Technology, Guangzhou, 510006 P.R. China; 4 College of Information Science and Technology, Jinan University, Guangzhou, 510632 P.R. China; 5 International Institute for Translational Chinese Medicine, Guangzhou University of Chinese Medicine, Guangzhou, 510006 P.R. China

## Abstract

Drug resistance caused by excessive and indiscriminate antibiotic usage has become a serious public health problem. The need of finding new antibacterial drugs is more urgent than ever before. Tyrosyl-tRNA synthase was proved to be a potent target in combating drug-resistant bacteria. In silico methodologies including molecular docking and 3D-QSAR were employed to investigate a series of newly reported tyrosyl-tRNA synthase inhibitors of furanone derivatives. Both internal and external cross-validation were conducted to obtain high predictive and satisfactory CoMFA model (*q* ^2^ = 0.611, *r* ^2^_*pred*_ = 0.933, *r* ^2^_*m*_ = 0.954) and CoMSIA model (*q* ^2^ = 0.546, *r* ^2^_*pred*_ = 0.959, *r* ^2^_*m*_ = 0.923). Docking results, which correspond with CoMFA/CoMSIA contour maps, gave the information for interactive mode exploration. Ten new molecules designed on the basis of QSAR and docking models have been predicted more potent than the most active compound 3-(4-hydroxyphenyl)-4-(2-morpholinoethoxy)furan-2(5H)-one (15) in the literatures. The results expand our understanding of furanones as inhibitors of tyrosyl-tRNA synthase and could be helpful in rationally designing of new analogs with more potent inhibitory activities.

## Introduction

Infectious diseases caused by bacteria are known as one of the most life-threatening health problem all over the world, whose chemotherapy using antimicrobial agents and antibiotics has been a critical public health tool for nearly a century, saving millions of lives around the world^1^. However, due to the indiscriminate usage of antibiotics in particular, surviving bacteria have evolved resistance against several antibiotics in recent decades^2^. Especially with the growth of multidrug resistance in bacteria, finding new antibacterial drugs becomes increasingly crucial in global researches^3^. Interruptions of protein synthesis have long been recognized as an attractive target of anti-bacterium, and its crucial enzyme aminoacyl-tRNA (aaRS) involved in protein synthesis catalyzed the bond between specific amino acid and its cognate tRNAs^4^. Hence, an increasing number of researches focused on aaRS inhibitors as a potent antibacterial agent. The inhibitors of leucyl-tRNA synthase of icofungipen and AN-2690 are both in clinical development for the treatment of onychomycosis^5^. Mupirocin, an inhibitor of isoleucyl-tRNA, shows good effect on infectious diseases as antibacterial agents^6^. Recently, a series of furan-2(5H)-one derivatives have been found remarkable inhibitory activities against tyrosyl-tRNA synthase^5^.

The three-dimensional quantitative structure-activity relationship (3D-QSAR) models, calculated via the most widely used comparative molecular field analysis (CoMFA)^7^ and comparative molecular similarity indices analysis (CoMSIA)^8^ incorporating the information of conformation or spatial orientation of molecules, are used for the rational design of most potent novel inhibitors. In this study, they were used to extract the structural features favored for tyrosyl-tRNA synthase inhibitors based on the skeleton of furan-2(5H)-one. The best model, which was developed from a dataset consisted of a 44 molecule training set and a 8 molecule test set, have been validated appropriately, and 10 novel compounds designed on the basis of the model have been predicted better activity than compound 15, the most active molecule in the literatures^5, 9^. Therefore, the established 3D-QSAR models of fifty-two molecules by CoMFA and CoMSIA could not only give the key structure requirements for the antimicrobial activity but also serve as a helpful guidance in design of novel antibiotics, especially for the control of drug-resistant superbugs.

## Methodology

### Methods and Data sets

Fifty-two furan-2(5H)-one tyrosyl-tRNA synthase inhibitors were collected from papers published by a certain research group^5, 9^. The biological data of the compounds and their structures were showed in Table 1. The pIC_50_(−logIC_50_) values converted from the origin IC_50_ was required as dependent variables in 3D-QSAR analysis. The pIC_50_ values covering 3 log units were considered as a homogenous and wide range dataset for 3D-QSAR studies^10^

**Table 1:** Furanone derivatives and their observed and predicted TS inhibition activities.

						
Compd.	pIC_50_	R^1^	R^2^	R^3^	Predicted Activity (CoMFA)	Residual	Predicted Activity (CoMSIA)	Residual
1	4.54	H	H		4.623	0.0867	4.28	−0.2564
2	4.14	H	H		4.574	0.4303	4.339	0.1961
3	4.06	H	H		4.494	0.4356	4.108	0.0491
4	4.27	H	H		4.477	0.2064	4.465	0.1946
5	4.45	H	H		4.875	0.4255	4.554	0.1042
6	5.21	H	H		5.465	0.2574	5.054	−0.1538
7	5.07	H	H		5.394	0.3286	4.929	−0.1365
8	4.69	H	Cl		4.867	0.1786	4.846	0.1579
9	4.61	H	Br		4.735	0.1206	4.908	0.2937
10	4.43	H	OMe		4.577	0.1509	4.091	−0.3348
11	6.21	Cl	H		6.508	0.3002	6.044	−0.1639
12	5.62	Br	H		6.467	0.847	5.809	0.1889
13^a^	4.48	OMe	H		4.341	−0.139	4.854	0.37
14	4.59	H	OH		4.996	0.4039	4.593	0.0012
15	7.00	OH	H		6.941	−0.059	7.002	0.002
		
		**R** ^**4**^	**R** ^**5**^					
16	4.07	H			4.321	0.2486	4.385	0.3125
17	4.32	H			4.4	0.0798	4.344	0.0235
18	4.39	H			4.527	0.1403	4.237	−0.1497
19	4.89	H			5.154	0.2609	4.828	−0.0652
20^a^	4.59	H			5.163	0.5782	4.387	−0.198
21	4.75	H			4.643	−0.1088	4.811	0.0589
22^a^	4.78	H			4.497	−0.2781	4.668	−0.1065
23	4.00	H			4.175	0.1754	4.619	0.6192
24	4.25	H			4.659	0.4138	4.428	0.1825
25^a^	4.74	H			4.821	0.081	4.901	0.161
26	4.38	H			4.595	0.2121	4.276	−0.1069
27	4.19	H			4.322	0.1329	3.974	−0.2149
28	5.09	H			5.268	0.1764	4.811	−0.2807
29	5.37	H			5.378	0.0115	5.301	−0.0653
30	4.95	H			5.083	0.1322	5.229	−0.0549
31^a^	4.78	H			4.501	−0.2811	4.879	0.1291
32^a^	5.28	H			4.3	−0.9839	4.41	−0.8744
33	4.48	H			4.96	0.4838	4.839	0.3631
34	4.30	H			4.744	0.4447	4.242	−0.057
35	4.12	H			4.598	0.4745	4.142	0.0189
36^a^	5.17	H			5.102	−0.068	4.788	−0.3820
37	4.03	H			4.226	0.1975	3.845	−0.1834
38	4.32	H			4.3	−0.0183	4.021	−0.2969
39	4.00	H			4.41	0.4101	4.129	0.1291
40	4.08	H			4.352	0.2754	4.147	0.0705
41	5.96	Cl			6.069	0.1103	5.549	−0.4096
42	5.46	Cl			5.663	0.2067	5.561	0.1046
43	5.10	Cl			5.253	0.1505	5.086	−0.0163
44	5.24	Cl			5.449	0.2049	5.318	0.0736
45	5.03	Cl			5.471	0.4393	5.293	0.2619
46^a^	4.78	Cl			5.265	0.4874	4.973	0.1956
47	6.05	Cl			6.085	0.0396	5.773	−0.273
48	4.75	H			5.065	0.3153	4.879	0.1291
49	5.13	H			5.348	0.2228	4.903	−0.2223
50	5.06	H			5.162	0.106	4.875	−0.1803
51	4.00	H			4.223	0.2232	4.036	0.0357
52	4.75	H			5.065	0.3153	4.879	0.1291

^a^Test set molecules.

. Eight molecules of structural variety and broad range of activity in the data were randomly chosen as test set to assess predictive ability of the resulting models, and therefore the remaining forty-four molecules were selected as a training set to generate the 3D-QSAR models.

### Molecular modeling and alignment

**Figure 1:**
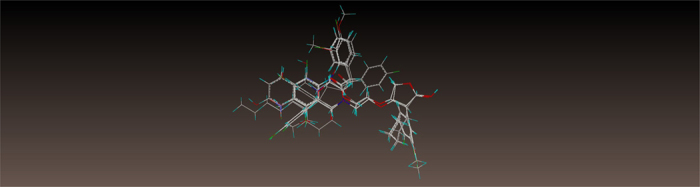
Molecular alignment.

The 3D structures of the furan-2(5H)-ones were build in SYBYL 8.1 (Tripos, Inc, St. Louis, MO, USA) molecular modeling package. The force field of standard Tripos molecular mechanics along with Gasteiger-Hűckel charge were employed to perform structure energy minimization^11^. The quality of molecular alignment was considered as a key factor for the robustness and predictive power of CoMFA and CoMSIA models^4, 12^. Here we applied molecular alignment to align all the molecules by using furan ring as the common skeleton and the most active compound 15 as the template molecule. To ensure the energy level of the conformations was reasonable, a further conjugate gradient method minimization of compound 15 was conducted and the convergence was reached. The aligned molecules were shown in Fig. 1.

### CoMFA and CoMSIA analysis

The descriptor fields of both methods were calculated in a three-dimensional cubic with one angstrom grid spacing. The frontier of the box extended extra 4 angstrom units from the border of aligned structures in each direction.

For CoMFA method, incorporating steric and electrostatic fields^13^, the the probe atom of a charged sp^3^ hybridized carbon atom was applied to compute electrostatic and steric fields. The cutoff value was 30 kcal∙mol^−1^ ^14^. As to CoMSIA approach, three extra fields including hydrogen bond acceptor, hydrogen bond donor and hydrophobic were considered^15^. A Gaussian function was also applied in calculating the similarity indices, making it accounts for all grid points^16^. The equation (1) for the similarity indices calculation is as below:

1 $${A}_{F,k(j)}^{q}=\sum {W}_{probe,k}{W}_{ik}{e}^{-\alpha {r}_{iq}^{2}}$$

where *A* ^*q*^ means the similarity index of point *q*, and *k* represent the physicochemical properties of electrostatic and steric descriptors; W_*probe,k*_ means the probe atom and the attenuation factor is a default value of 0.3; *i* means summation index of the molecule *j*, and W_*ik*_ is the observed value *k* of a specific property of the atom *i* ^17^.

### Partial least squares (PLS) analysis

In order to build a statistically significant model, PLS method^18^ was introduced to correlate the both fields to the pIC_50_ values linearly. Leave-one-out (LOO) method was performed first in the cross-validation in which each compound is deleted from the dataset and the activity of the “leave-one” molecule is predicted using the model build based on the remaining molecules in the dataset^19^. With the default value of column filtering at 2.0 kcal∙mol^−1^, the optimum number of components (ONC) was obtained on the lowest standard error of prediction (SEP), which is usually corresponds to the highest cross-validated squared coefficient (*q* ^2^). To avoid the model over-fit, higher numbers of component will not be accepted unless the *q* ^2^ could have risen by 10% or more^20^. Non-cross-validation was then conduct to build the final 3D-QSAR models. The correlation coefficient of LOO method (*r* ^2^_*cv*_) was defined by equation (2) as follows:

2 $${r}_{cv}^{2}=1-\sum {({Y}_{obs}-{Y}_{pre})}^{2}/\sum {({Y}_{obs}-{Y}_{mean})}^{2}$$

where Y_mean_, Y_pre_ and Y_obs_, means average, calculated and actual pIC_50_, respectively^21, 22^.

### Sensitivity of a PLS model

Most molecules of the data set may have “twins”, which make a near twin of each left-out molecule likely remain in the training data and lead to a good prediction, therefore, the *q* ^2^ statistic may give you a false sense of confidence. Progressive scrambling was often used to determine the sensitivity of a QSAR model to small systemic perturbations of the response variable for the model’s stability^23, 24^.

### Predictive correlation coefficient (*r*^2^_*pred*_)

Normally *q* ^2^ is considered as a productive but not sufficient parameter in validating the model. Sometimes models with high *r* ^2^_*cv*_ and *r* ^2^ values may be improper in many cases. The external predictive correlation coefficient (*r* ^2^_*pred*_)^25^ was calculated to estimate the predict ability. The predictive correlation coefficient (*r* ^2^_*pred*_) was calculated by the following equation (3):

3 $${{r}^{2}}_{pred}=({\rm{SD}}-{\rm{PRESS}})/{\rm{SD}}$$

where, SD is the sum of the squared deviations between the activity values of the test set and mean activities of the training molecules; PRESS stands for the sum of squared deviations between calculated and observed activity values for each structure of the test set^26, 27^.

### Molecular docking and MD simulation

Molecular docking technique is an important method in discovering novel small-molecule drugs^28–32^. In our study, Surflex-Dock was used to perform the molecular docking. Tyrosyl-tRNA synthase’s crystal structure 3P0H which contains a ligand was obtained from the PDB database^33^. The hypothetical protomol was used to probe steric and electrostatic interactions of the active pocket. H-bond acceptor and donor substituent as well as hydrophobic fields were also investigated^34, 35^. Before docking, hydrogen atoms of the receptor were added first, and the Kollman-All force field was applied in the prepared structures^36–38^. The definition of active site definition was performed based on the original ligand in the crystal. We chose compound 15 as the subject to dock into the active pocket under the conditions previously optimized. As to the newly designed molecules, the docking study along with MD simulation was also conducted to validate the calculated activities and the interaction mechanisms. The docking method was similar as the docking process above and the binding conformations with highest score were subjected to MD simulations in AMBER molecular dynamics package^39^. Each MD time was 10 ns in explicit solvent. Antechamber tool was applied to generate the partial atomic charges of each molecule and the force field ff12SB was loaded to the receptor. Truncated octahedral water box by the TIP3P water model was chosen to encompass the complexes before minimization and MD. After the MD, analysis was performed with the ptraj analysis tool based on the last 2 ns trajectories of the simulation. The binding free energies of complexes were calculated using the MM-PBSA^40^.

## Results and Discussion

### CoMFA and CoMSIA results

**Figure 2:**
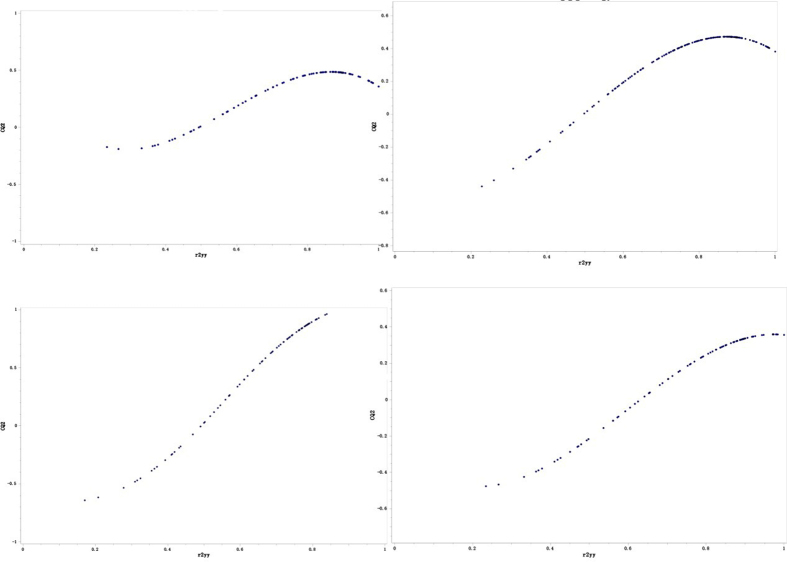
Variation fitted curves for progressive scrambling analyses with random number seed: (left upper) 4 components; (right upper) 5 components; (left lower) 6 components; (right lower) 7 components.

**Figure 3:**
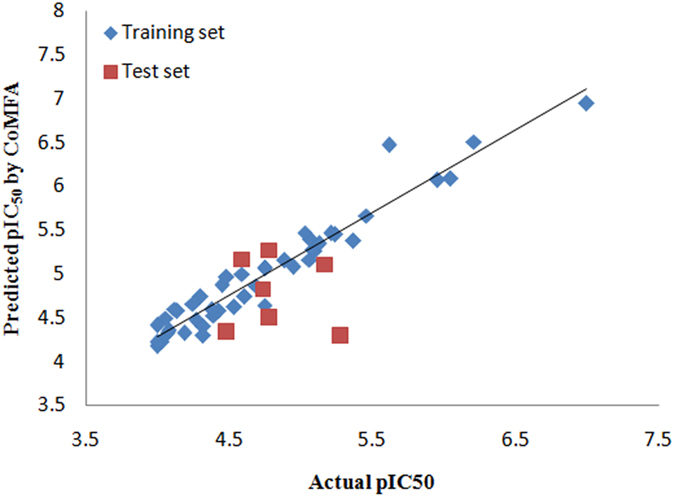
Scatter plots of predicted versus actual pIC_50_ of CoMFA model.

**Figure 4:**
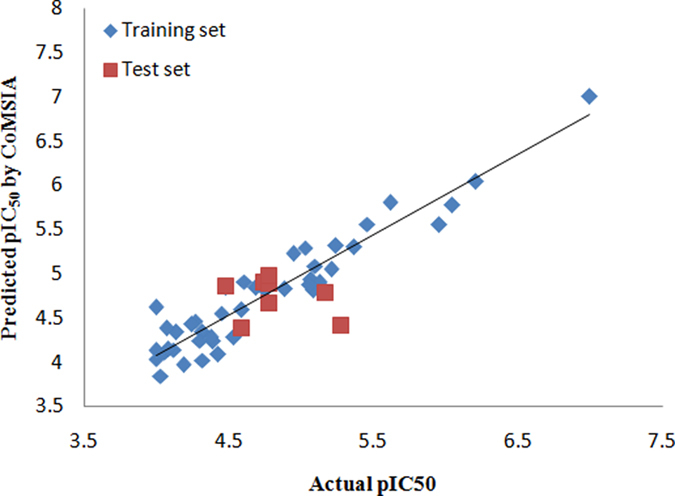
Scatter plots of predicted versus actual pIC_50_ of CoMSIA model.

Both models were obtained on the basis of a 44 molecules training set of whose pIC_50_ values ranging from 4 to 7. Table 2 listed the statistical parameters of CoMFA and CoMSIA models. And the results of the progressive scrambling demonstrated that the value for the slope in the five component model is admissible (Fig. 2), and with a minimum cSDEP and maximum*Q* ^2^ the optimum statistics are also seen for six components in Table 3. The cross-validated correlation coefficient (*q* ^2^) of CoMFA model based on both electrostatic and steric fields is 0.611 (>0.5). Other parameters such as ONC as 6, *S*EE (standard error estimate) of 0.179, non-cross-validated correlation coefficient (*r* ^2^) of 0.940, standard error estimate (*S*) of 0.179, F value of 96.577 and predictive correlation coefficient (r^2^_pred_) of 0.759 was derived. The proportion of electrostatic and steric fields contributes 0.281 and 0.719. CoMSIA with an ONC of 8 gave a *q* ^2^ of 0.577 (>0.5), *r* ^2^ of 0.916, *S*EE of 0.212, F value of 67.037 and *r* ^2^_pred_ of 0.791. Contributions proportion of electrostaticsteric, steric electrostatic, hydrogen H-bond donor and hydrogen acceptor contributes were 0.152, 0.525, 0.221 and 0.101, respectively. The correlations between the calculated and actual pIC_50_ values of the whole data set were listed in Figs 3 and 4. These PLS statistics revealed that the our proposed CoMFA and CoMSIA models could adequately predict all the compounds in the test set.

**Table 2:** Statistical results.

PLS Statistics	CoMFA	CoMSIA
*q* ^2^	0.611	0.546
*r* ^2^	0.940	0.905
S	0.179	0.232
F	96.577	41.575
*r* ^2^_bootstrap_	0.954	0.923
S_bootstrap_	0.160	0.193
Optimal Components	6	8
Field Distribution %		
Steric	71.9	15.2
Electrostatic	28.1	52.5
Hydrophobic		
Hydrogen Bond Donor		22.1
Hydrogen Bond Acceptor		10.1
*r* ^2^_pred_	0.759	0.791

**Table 3:** Progressive scrambling results of the CoMFA model.

Components	*Q* ^2^	cSDEP	d*q* ^2^/d*r* ^2^_yy'_
4	0.458	0.524	1.267
5	0.469	0.533	1.227
6	0.541	0.564	1.015
7	0.419	0.571	1.355

### CoMFA contour map analysis

**Figure 5:**
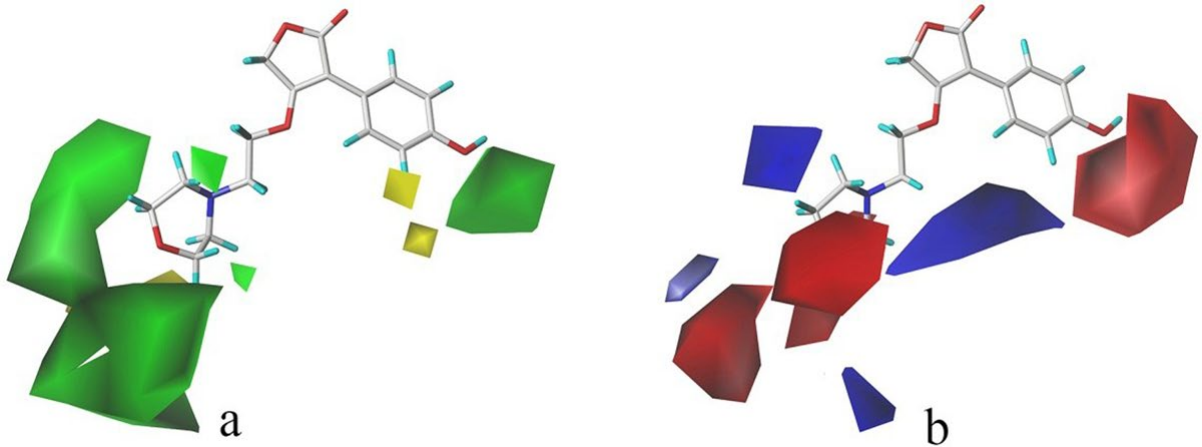
CoMFA contour maps with compound 15 as template. (**a**) Steric: green contours indicates bulky groups favored, yellow contours means bulky groups disfavored. (**b**) Electrostatic fields: blue contours stand for electron-donating groups favored ragion, red contours located where electron-withdrawing groups favored.

CoMFA contour maps were drawn vividly to explore the areas in three-dimensional space around the compounds where modifications would alter activity. The contour maps are revealed in Fig. 5, with compound 15 as the template molecule. In Fig. 5a, the green blocks mean a bulky group favored area, while the yellow blocks indicate that minor substituent are preferable to enhance the activity. In Fig. 5b, the electron-donating group and electron-withdrawing group favored region are represented by blue and red contours, respectively.

As shown in Fig. 5a, a giant green block located near the hydroxyl group substituted to phenyl ring along with the large green contours around the non-aromatic hexatomic ring of compound 15 indicate that bulky groups here can increase the activity. These factors may explain why compounds 11, 12, 41, 42 and 47 with halogen substituents in this area are more potent than molecules without any substituents at this particular position. The yellow contour on the top or bottom of the non-aromatic hexatomic ring of compound 15 suggested the unfavorable influence of bulky groups. This might be the reason why compound 40 whose methoxyl substituent is not on the plane of the molecule showed significantly decreased activities.

As shown in Fig. 5b, red contours around substituent group R^1^ or R^4^, and *para*- and *meta*-positions of hexatomic ring of group R^3^ or R^5^ revealed that electron-withdrawing substituents were considered to be beneficial to the activity. The compounds 29, 41, 42 and 47, with electron-withdrawing groups at R^1^ and the *meta*-position of hexatomic ring of group R^3^ or R^5^, displayed the good bioactivity. On the contrary, the blue contour around *meta*-position of hexatomic ring of group R^3^ or R^5^ told us that electron-donating groups here would be expected. Therefore, compound 28 with the methyl group at the *meta*-position of benzene ring displayed good IC_50_ values.

### CoMSIA contour map analysis

**Figure 6:**
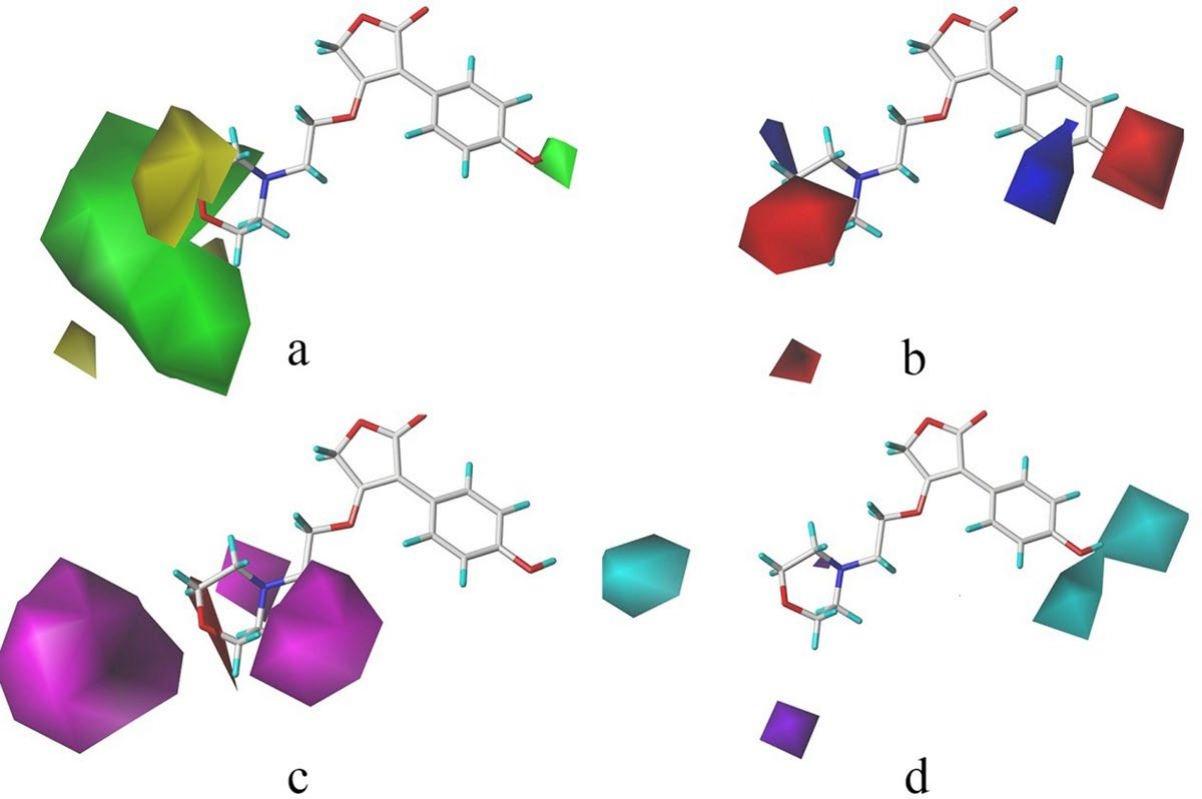
CoMSIA contour maps with compound 15 as template. (**a**) Steric: Green contours favored bulky regions, and yellow contours bulkily disfavored regions. (**b**) Electrostatic: Red contours indicates electron-withdrawing groups favored, and blue contours means electron-donating groups favored. (**c**) H-bond acceptor: Magenta and red contours indicate h-bond acceptor favorable and unfavorable respectively. (**d**) H-bond donor: Cyan and purple contours stand for favorable and unfavorable respectively.

CoMSIA contour maps of steric, electrostatic, H-bond donor and acceptor field are revealed in Fig. 6. Generally the CoMSIA contour maps of electrostatic and steric field are similar to CoMFA models. However, the electrostatic field in CoMSIA model appended a blue contour around group R^2^, which means that electron-donating substituents were also preferred in this region.

The hydrogen-bond donor field was presented in Fig. 6c. Cyan contour around hydroxyl substituent of compound 15 revealed that hydrogen bond donor was preferred in this region. In hydrogen bond acceptor field (Fig. 6d), the purple contour was around group R^3^, which revealed that hydrogen acceptor was preferred in this region.

Therefore, compounds 6 and 11 with hydrogen acceptor at group R^3^ showed good IC_50_ values. Furthermore, the hydrogen acceptor was also preferred in the side chain of furan ring.

### Molecular Docking Analysis

**Figure 7:**
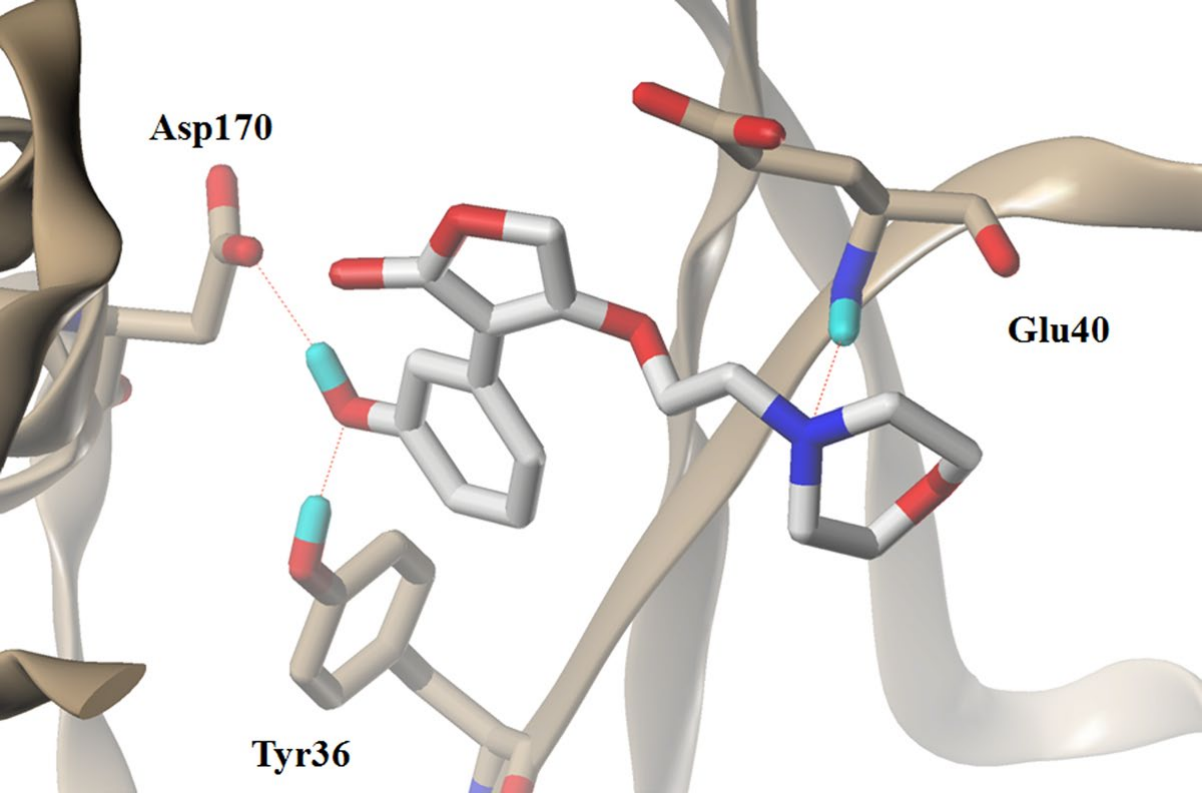
The docking result of compound 15 and tyrosyl-tRNA synthase: H-bond between compound 15 and residues.

**Figure 8:**
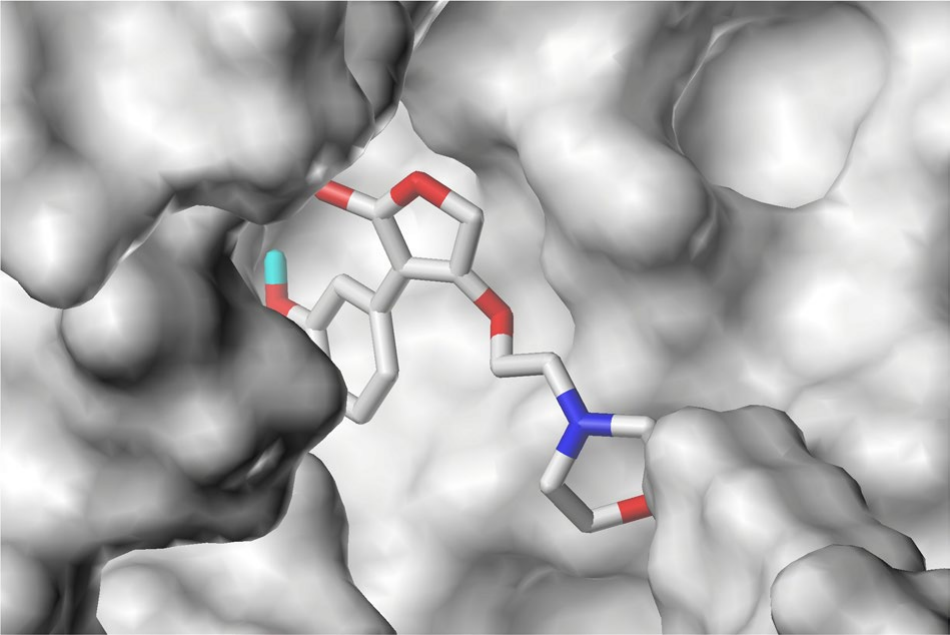
The docking result of compound 15 and tyrosyl-tRNA synthase: Compound 15 in the binding pocket.

The Figs 7 and 8 demostrated the binding modes of tyrosyl-tRNA synthase with compound 15. The hydroxyl groups at R^1^ position formed H-bonds with Tyr46 and Asp170 residue by acting as H-bond acceptor and donor simultaneously. The morpholine nitrogen in R^3^, which served as a H-bond acceptor, also formed a hydrogen bond with Glu40. These interactions summarized from Fig. 7 coincided satisfactorily with the contour maps derived from CoMSIA method above. Moreover, as can be seen in Fig. 8, a cavity located beneath the morpholine ring where steric contour map hints us a bulky group favored verified our results.

### Summary of the structure-activity relationships

**Figure 9:**
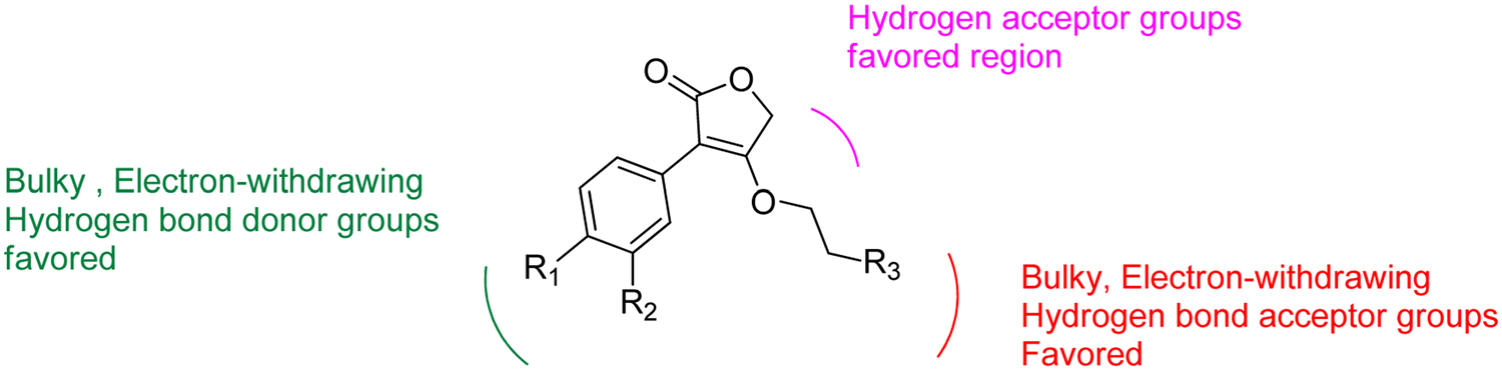
SAR summarized based on our work.

The SAR derived from the present work were illustrated in Fig. 9. To be specific, the bulky, electron-withdrawing and h-bond donor groups such as hydroxyl group at R^1^ or R^4^ position are essential to improve the antibacterial activity. The electron-withdrawing, H-bond acceptor or bulky groups like ether bond at *para*-position or *meta*-position of substituent group R^3^ or R^5^ may increase their activities. Introducing electron-donating groups at *meta*-position of substituent group R^3^ or R^5^ may also enhance the activity. Furthermore, the hydrogen acceptor was also preferred in the side chain at furan ring.

### Design of Novel Derivatives

After gaining the SAR revealed by the foregoing study, ten new furan-2(5H)-ones (D1-D10) were designed and predicted. Molecular alignment was conduct on these new molecules and their activities were calculated to be better than compound 15 (pIC_50_ = 6.941). Such results indicated that these 3D-QSAR models with considerable predictive ability could be prospectively used in structure modification and optimization. The structures and calculated pIC_50_ of these newly designed molecules were listed in Table 4

**Table 4:** Structures and calculated pIC_50_ of the designed molecules.


Molecule	Substituent			Predicted pIC_50_	
	R1	R2	R3	CoMFA	CoMISA
15	OH	H		6.941	7.002
D1	OH	H		7.614	7.256
D2	OH	H		8.021	7.995
D3	OH	H		8.678	8.517
D4	OH	OH		8.624	8.951
D5	OH	OH		8.579	8.544
D6	CONH_2_	OH		9.025	9.173
D7	CONH_2_	OH		9.647	9.815
D8	CONH_2_	OH		8.957	9.016
D9	CONH_2_	OH		8.166	8.527
D10	CONH_2_	OH		8.647	8.928

.

### Validation of Newly-designed Derivatives

**Figure 10:**
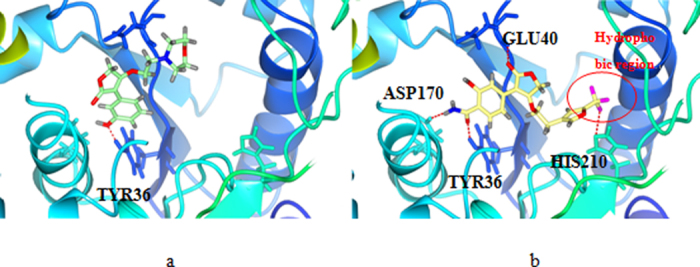
Binding conformations of compound 15 (**a**) and D7 (**b**).

**Figure 11:**
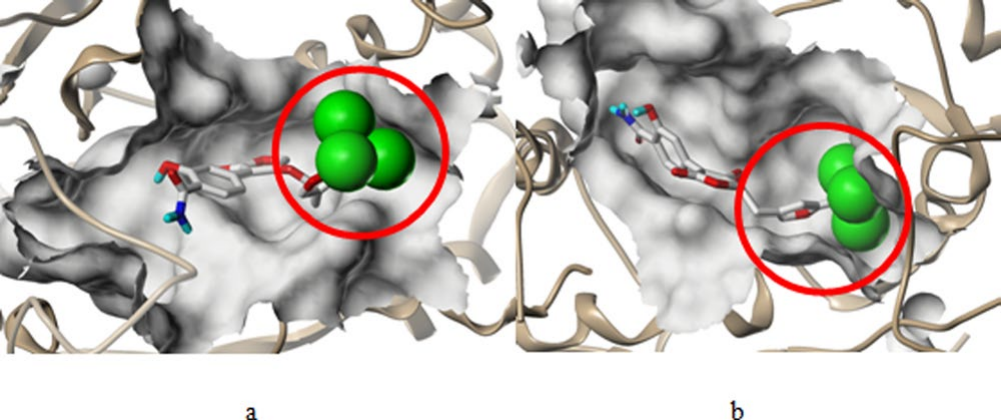
Binding conformations of compound D6 after optimizing (**a**) and before optimazing (**b**), chlorineatoms are shown in spacefill form.

These ten new furan-2(5H)-ones (D1-D10) were re-docked to the receptor 3P0H and based on which MD simulations were also performed to check the potencies by assess the binding affinity and free energy. The docking results was listed in the following Table 5. The ΔG calculated from the MD simulation results were also shown below. Compound D7 showinged significant affinity and the interactions between ligand and receptor were depicted in Fig. 10, where compound 15 was also exhibited as the reference. We can find that more hydrogen bonds with stronger bond energy formed based on the modified group, for instance the acylamino in R^1^ connecting Asp170 with a new hydrogen bond and interacting Tyr36 with a hydrogen bond of −3.3 K cal/mol is more favored than the original hydroxyl in this position whose hydrogen bond was −2.9 K cal/mol. However, comparing with the predicted pIC_50_ values, compounds with trichloromethyl or bromine demonstrated less potency, especially the compound D6 are of poor affinity. By analyzing the conformations optimized from the MD simulation, we found that although the substituent group of R^3^ are bulky favored, the group size is not unlimited, substituent group larger than furan ring with trifluoromethyl may cause crash of protein and ligand thus changed the equalized conformation after the MD simulation. Compound D1 suffered the same situation that showed dissatisfactory inhibitory activity in the docking and optimization. In Fig. 11, the conformation of compound D6 altered hugely to extend the trifluoromethyl out of the surface to reduce the tension. Additionally the amide group in R^1^ served as a H-bond donor by forming a stable hydrogen bond with His210 and verified the SAR obtained above. According to the docking and MD simulation study the compound D7 was also proven to be most promising in tyrosyl-tRNA synthase inhibition.

**Table 5:** Docking scores and calculated*ΔG* of designed compounds.

Molecule	Docking score	*ΔG* (kcal · mol^−1^)
D1	6.4131	−19.4
D2	8.0254	−32.4
D3	6.8264	−29.9
D4	7.5216	−30.2
D5	7.1824	−24.7
D6	6.3483	−22.1
D7	8.4677	−34.5
D8	7.1582	−21.1
D9	6.9573	−27.8
D10	7.0564	−19.4

## Conclusion

The tyrosyl-tRNA synthase inhibitors provide a promising approach in fighting against drug-resistant bacteria. In our present studies, we have established CoMFA model (*q* ^2^  = 0.611, *r* ^2^ = 0.940) and CoMSIA model (*q* ^2^ = 0.546, *r* ^2^ = 0.905) with satisfactory correlation and predictive abilities from fifty-two tyrosyl-tRNA synthase inhibitors. The CoMFA and CoMSIA contour maps provided information to summarize the SAR and characterized key features affecting the antibacterial activity. Moreover, the prediction ability of the model validated by the test set turned out to be satisfactory which means these models could be applied in predicting the activities of new compounds. Thus 10 new molecules were designed and predicted to be more potent than compound 15, these results were further validated by MD simulation study.All the work above confirmed that our models can provide a ponderable clue in designing novel antimicrobial agents.

## References

[1] Garciaalvarez L, Dawson S, Cookson B, Hawkey P (2012). Working across the veterinary and human health sectors. J Antimicrob Chemother.

[2] Estephane J (2008). N-Acyl-3-amino-5H-furanone derivatives as new inhibitors of LuxR-dependent quorum sensing: Synthesis, biological evaluation and binding mode study. Bioorg Med Chem Lett.

[3] Chaudhary AS (2016). A review of global initiatives to fight antibiotic resistance and recent antibiotics’ discovery. Acta Pharmaceutica Sinica.

[4] Yanagisawa T, Sumida T, Ishii R, Takemoto C, Yokoyama S (2010). A paralog of lysyl-tRNA synthetase aminoacylates a conserved lysine residue in translation elongation factor P. Nat Struct Mol Biol.

[5] Xiao ZP (2011). 4-Alkoxy-3-arylfuran-2(5H)-ones as inhibitors of tyrosyl-tRNA synthetase: Synthesis, molecular docking and antibacterial evaluation. Bioorg Med Chem Lett.

[6] Vijver PVD (2008). Aminoacyl-tRNA Synthetase Inhibitors as Potent and Synergistic Immunosuppressants. J Med Chem.

[7] Cramer RD, Patterson DE, Bunce JD (1988). Comparative molecular field analysis (CoMFA): 1. Effect of shape on binding of steroids to carrier proteins. J Am Chem Soc.

[8] Klebe G, Abraham U, Mietzner T (1994). Molecular similarity indices in a comparative ananlysis (CoMSIA) of drug molecules to correlate and predict their biological activity. J Med Chem.

[9] Wang XD (2013). 3-Aryl-4-acyloxyethoxyfuran-2(5H)-ones as inhibitors of tyrosyl-tRNA synthetase: synthesis, molecular docking and antibacterial evaluation. Bioorg Med Chem.

[10] Cramer RD, Patterson DE, Bunce JD (1989). Recent advances in comparative molecular field analysis (CoMFA). Prog Clin Biol Res.

[11] Ravichandran V, Sankar S, Agrawal RK (2009). Predicting anti-HIV activity of 1,1,3-trixox [1,2,4]-thiadiazine (TTD) derivatives: 3D QSAR approach. Med Chem Res.

[12] Zheng J (2011). Exploring QSARs for 5-lipoxygenase (5-LO) inhibitory activity of 2-substituted 5-hydroxyindole-3-carboxylates by CoMFA and CoMSIA. Chem Biol Drug Des.

[13] Song QL, Sun PH, Chen WM (2010). Exploring 3D-QSAR for ketolide derivatives as antibacterial agents using CoMFA and CoMSIA. Lett Drug Des Discov.

[14] Hu R, Barbault F, Delamar M, Zhang R (2009). Receptor- and ligand-based 3D-QSAR study for a series of non-nucleoside HIV-1 reverse transcriptase inhibitors. Bioorg Med Chem.

[15] Klebe G, Abraham U (1999). Comparative molecular similarity index analysis (CoMSIA) to study hydrogen-bonding properties and to score combinatorial libraries. J Comput Aided Mol Des.

[16] Zheng J (2014). Insight into the interactions between novel isoquinolin-1,3-dione derivatives and cyclin-dependent kinase 4 combining QSAR and molecular docking. Plos One.

[17] Politi A (2009). Application of 3D QSAR CoMFA/CoMSIA and in silico docking studies on novel rennin inhibitors against cardiovascular diseases. Eur J Med Chem.

[18] Bush BL, Jr NR (1993). Sample-distance partial least squares: PLS optimized for many variables, with application to CoMFA. J Comput Aided Mol Des.

[19] Lu XY (2010). Molecular docking-guided 3D-QSAR studies of substituted isoquinoline-1,3-(2H, 4H)-diones as cyclin-dependent kinase (CDK4) inhibitors. J Mol Model.

[20] Zhang N, Zhong R (2010). Docking and 3D-QSAR studies of 7-hydroxycoumarin derivatives as CK2 inhibitors. Eur J Med Chem.

[21] Sun J, Cai S, Yan N, Mei H (2010). Docking and 3D-QSAR studies of influenza neuraminidase inhibitors using three dimensional holographic vector of atomic interaction field analysis. Eur J Med Chem.

[22] Puntambekar DS, Giridhar R, Yadav MR (2006). Understanding the antitumor activity of novel tricyclicpiperazinyl derivatives as farnesyltransferase inhibitors using CoMFA and CoMSIA. Eur. J Med Chem Res.

[23] Clark RD, Fox PC (2004). Statistical variation in progressive scrambling. J Comput Aided Mol Des.

[24] Luco JM, Ferretti FH (1997). QSAR based on multiple linear regression and PLS methods for the anti-HIV activity of a large group of HEPT derivatives. J Chem Inf Comput Sci.

[25] Golbraikh A, Tropsha A (2002). Beware of q2!. J Mol Graph Model.

[26] Cichero E, Cesarini S, Mosti L, Fossa P (2010). CoMFA and CoMSIA analyses on 4-oxo-1,4-Dihydroq-uinoline and 4-oxo-1, 4-dihydro-1, 5-, -1, 6- and -1, 8-naphthyridine derivatives as selective CB2 receptor agonists. J Mol Model.

[27] Park CHY (2011). A comparative study of quantitative structure activity relationship methods based on antitumor diarylsulfonylureas. Eur J Med Chem.

[28] Wang J, Kollman PA, Kuntz ID (1999). Flexible ligand docking: a multistep strategy approach. Proteins.

[29] Jain AN (2003). Surflex: Fully automatic flexible molecular docking using a molecular similarity-based search engine. J Med Chem.

[30] Jain AN (2007). Surflex-Dock 2.1: Robust performance from ligand energetic modeling, ring flexibility, and knowledge-based search. J Comput Aided Mol Des.

[31] Spitzer GM, Wellenzohn B, Laggner C, Langer T, Liedl KR (2007). DNA minor groove pharmacophores describing sequence specific properties. J Chem Inf Model.

[32] Ambure PS, Gangwal RP, Sangamwar AT (2012). 3D-QSAR and molecular docking analysis of biphenyl amide derivatives as p38 alpha mitogen-activated protein kinase inhibitors. Mol Divers.

[33] Larson ET (2011). The double-length tyrosyl-tRNA synthetase from the eukaryote Leishmania major forms an intrinsically asymmetric pseudo-dimer. J Mol Biol.

[34] Ruppert J, Welch W, Jain AN (1997). Automatic identification and representation of protein binding sites for molecular docking. Protein Sci.

[35] Holt PA, Chaires JB, Trent JO (2008). Molecular docking of intercalators and groove-binders to nucleic acids using Autodock and Surflex. J Chem Inf Model.

[36] Li YF (2016). Prediction and evaluation of thelipase inhibitory activities of tea polyphenols with 3D-QSAR models. Sci Rep.

[37] Muthas D, Sabnis YA, Lundborg M, Karlen A (2008). Is it possible to increase hit rates in structure-based virtual screening by pharmacophore filtering? An investigation of the advantages and pitfalls of post-filtering. J Mol Graph Model.

[38] van Westen GJ, Overington JP (2013). A ligand’s-eye view of protein similarity. Nat Methods.

[39] Case D. A., *et al*. AMBER 12, University of California, San Francisco (2012).

[40] Peng CK (2017). Novel 4-(4-substituted amidobenzyl)furan-2(5H)-one derivatives as topoisomerase I inhibitors. Eur J Med Chem.

